# Preliminary research in preparation for a randomized trial of brief intervention strategies for adolescents in school-based health centers.

**DOI:** 10.1186/1940-0640-10-S2-O20

**Published:** 2015-09-24

**Authors:** Jan Gryczynski, Robert P Schwartz, Shannon G Mitchell, Letitia Dzirasa, Kristi Dusek, Kevin O'Grady, Alexander Cowell

**Affiliations:** 1 Social Research Center, Friends Research Institute, Baltimore, USA; 2 Baltimore Medical System, Baltimore, USA; 3 RTI International, Research Triangle Park, USA

## Background

School-based health centers (SBHCs) offering a wide scope of health services are an expanding model of healthcare in the US. SBHCs are potential venues for brief intervention (BI), but little is known about the best way to deliver BI in these settings. The objective is to describe pilot data collected in preparation for a randomized trial of nurse- vs. computer-delivered BI for high school SBHC patients with risky alcohol or cannabis use.

## Material and methods

In preparation for the randomized trial, we (1) reviewed electronic medical record (EMR) data on patient volume and risk behaviors, and (2) conducted a longitudinal survey with an assessment-only cohort of 50 adolescents representing the target population for the forthcoming trial. Patients were eligible if they endorsed alcohol or cannabis use and scored positive on the CRAFFT, a brief substance use screening tool. Audio computer-assisted self-interviewing was used to assess lifetime and past 30 day frequency of substance use and sex risk behaviors, with follow-up assessments at 3- and 6-months.

## Results

In the year prior to initiating study activities, the two participating SBHCs logged 5,967 visits with 1,083 unique patients, of whom 11% had alcohol and/or drug use recorded in the EMR. Participants with risky substance use in the assessment-only cohort reported baseline rates of past 30-day cannabis and alcohol use of 88% and 50%, respectively. Base rates of past 30 day unprotected sex and sex while high/drunk were 44% and 34%, respectively. Follow-ups will examine the short-term natural trajectories of these behaviors.

## Conclusions

Our pilot data supports SBHCs as promising sites for delivering BIs to adolescents. Our forthcoming trial will examine the comparative effectiveness of two BI approaches at these SBHCs.

